# Supplementary material for: Adverse Reaction Profiles Related to Gastrointestinal Bleeding Events Associated with BCR-ABL Tyrosine Kinase Inhibitors
Source: Medicina (Kaunas). 2022 Oct 20;58(10):1495. doi: 10.3390/medicina58101495 (PMC9609656; doi:10.3390/medicina58101495)
Supplement: Supplementary file 1 [file medicina-58-01495-s001.zip › medicina-1908502-supplementary.pdf]

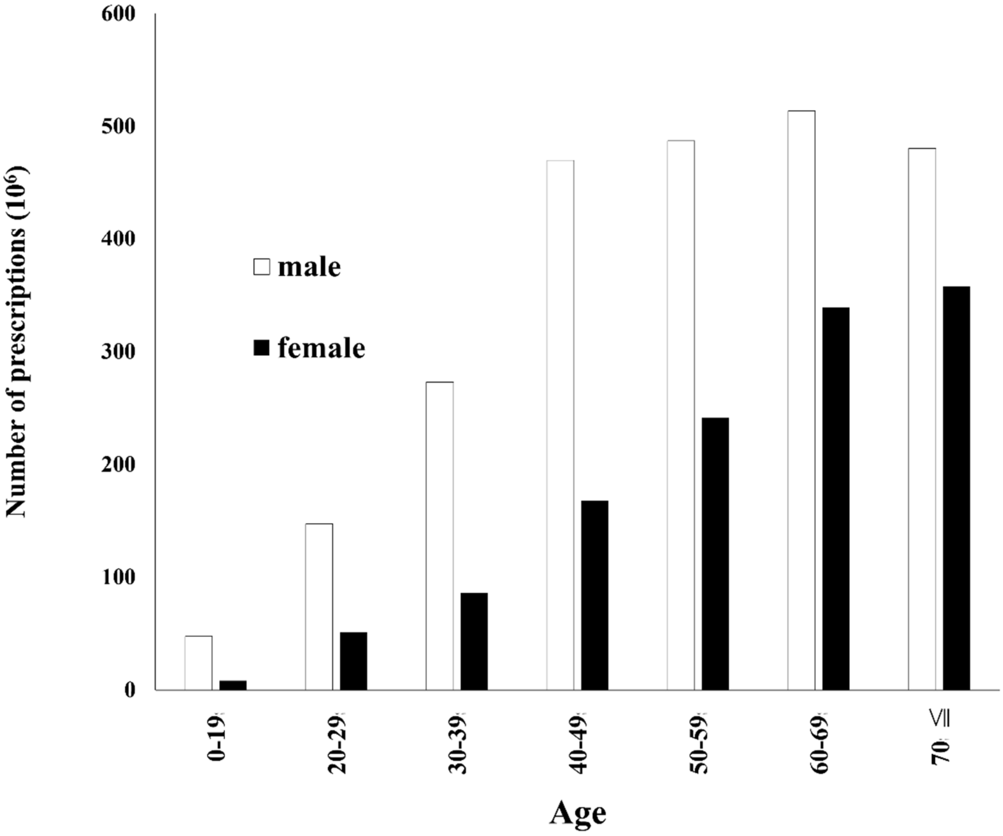

Supplementary Figure S1. Number of dasatinib prescriptions summarized according to patient age and sex. We determined the number of prescription drugs using the data from the 5th National Database of Health Insurance Claims and Specific Health Checkups of Open Data from 2018 (https://www.mhlw.go.jp/ndb/opendatasite accessed on December 31, 2021). The number of dasatinib prescriptions issued is shown according to age group and sex.

All values are $\times 10^6$

0–19 years: male 47.564; female 8.342

20–29 years: male 147.583; female 51.074

30–39 years: male 273.005; female 85.875

40–49 years: male 469.847; female 168.206

50–59 years: male 486.997; female 241.34

60–69 years: male 513.684; female 338.88

>70 years: male 480.341; female 357.647
